# Mechanisms of Abnormal Lipid Metabolism in the Pathogenesis of Disease

**DOI:** 10.3390/ijms25158465

**Published:** 2024-08-02

**Authors:** Linna Xu, Qingqing Yang, Jinghua Zhou

**Affiliations:** School of Basic Medicine Sciences, Hangzhou Normal University, Hangzhou 311121, China

**Keywords:** abnormal lipid metabolism, endocrine system diseases, cardiovascular system diseases, neurodegenerative diseases, cancer

## Abstract

Lipid metabolism is a critical component in preserving homeostasis and health, and lipids are significant chemicals involved in energy metabolism in living things. With the growing interest in lipid metabolism in recent years, an increasing number of studies have demonstrated the close relationship between abnormalities in lipid metabolism and the development of numerous human diseases, including cancer, cardiovascular, neurological, and endocrine system diseases. Thus, understanding how aberrant lipid metabolism contributes to the development of related diseases and how it works offers a theoretical foundation for treating and preventing related human diseases as well as new avenues for the targeted treatment of related diseases. Therefore, we discuss the processes of aberrant lipid metabolism in various human diseases in this review, including diseases of the cardiovascular system, neurodegenerative diseases, endocrine system diseases (such as obesity and type 2 diabetes mellitus), and other diseases including cancer.

## 1. Introduction

The complex physiological process of lipid metabolism is involved in the regulation of nutrients, hormones, and organismal homeostasis [1]. Abnormalities in lipids and their metabolites in the segments of synthesis, storage, and catabolism tend to alter plasma lipoprotein levels, structure, and function, which, in turn, cause the emergence and progression of related diseases [2]. The normal function of its various components is essential for maintaining the health of the body. In recent years, with the improvement of people’s living standards, dietary habits, and lifestyle changes, the incidence of lipid metabolism disorders and the incidence of many human diseases related to them have shown an increasing trend [3]. Being the most prevalent metabolic disease of the endocrine system, obesity is primarily caused by improper lipid metabolism and excessive caloric intake. Disorders related to lipid metabolism also exacerbate the development and occurrence of obesity. One of the common metabolic diseases affecting the endocrine system is type 2 diabetes mellitus (T2DM). Recent research has linked abnormalities in lipid metabolism to the onset and progression of the disease. Additionally, pathological studies have revealed that ectopic adipose tissue surrounds the β cells in the pancreatic islets, exacerbating the abnormalities in insulin secretion. Simultaneously, aberrant lipid metabolism also raises the risk of cardiovascular disease (CVD), particularly atherosclerosis and coronary heart disease [4,5]. It has been discovered that aberrant lipoprotein and polyunsaturated fatty acid metabolism contribute to the onset and progression of CVD, and an increasing number of studies has shown that abnormal lipid metabolism can either directly or indirectly raise the incidence of cardiovascular disease and mortality [6,7,8,9]. Anomalies related to the metabolism of lipids can impact not only the beginning and course of metabolic disorders but also the development and advancement of cancer. One of the defining characteristics of the development of cancer is abnormal intracellular lipid metabolism, whereby increased fatty acid (FA) de novo synthesis results in intracellular lipid accumulation in cancer cells, as well as increased levels of β-oxidation (intracellular oxidative stress) and, subsequently, more energy. Additionally, abnormal lipid accumulation occurs in the tumor microenvironment (TME), exacerbating tumor infiltration and further contributing to the creation of an immunosuppressive tumor microenvironment, which allows tumor cells to evade immune attack. Chronic disorders like neurodegenerative diseases, nonalcoholic fatty liver disease (NAFLD), chronic kidney disease (CKD), and osteoarthritis are also accompanied by abnormalities in lipid metabolism.

Thus, there is significant theoretical significance as well as actual clinical value in the thorough investigation of the role of aberrant lipid metabolism and its mechanism in the development of linked diseases. As a result, this study examines the contribution of aberrant lipid metabolism to the development of associated diseases, such as cancer, neurological disorders, cardiovascular diseases, and endocrine system disorders.

## 2. Abnormalities in Lipid Metabolism and Endocrine System Diseases

Lipid metabolism plays a key role in maintaining the homeostasis of the body. Abnormal lipid metabolism often leads to endocrine hormone disorders and further leads to obesity, diabetes, hyperglycemia, hyperlipidemia, and other endocrine diseases [10]. Obesity is a metabolic disease that causes excessive fat accumulation in the human body due to a variety of reasons and even leads to excessive weight gain and causes pathological and physiological changes. Endocrine-related metabolic disorders, such as obesity and diabetes, are caused by the dysregulation of lipid metabolism. Therefore, in order to provide new perspectives for future research on abnormal lipid metabolism and the occurrence and development of endocrine system disorders, this section focuses on the relationship between obesity, T2DM, and abnormal lipid metabolism.

### 2.1. Obesity and Abnormal Lipid Metabolism

Globally, obesity is becoming a serious health concern, and its incidence is increasing everywhere. The majority of the time, obesity is brought on by an imbalance between the body’s energy expenditure and intake [11]. This imbalance encourages the growth of white adipose tissue (WAT) in obese individuals, which, in turn, causes lipid metabolism abnormalities in the body. The body uses WAT as its primary adipose tissue for energy storage [12]. The big lipid droplets found in its cells include cholesterol ester (CE), triglycerides (TGs), and other lipids. Large amounts of free fatty acids (FFAs) are released during the hydrolysis of accumulated TGs in the WAT of obese patients. This leads to plasma FFA levels that are typically higher in obese patients [13,14], and the existence of large amounts of free fatty acids further exacerbates obesity [15]. This section primarily addresses the abnormal lipid metabolism process, its associated metabolites, and important enzymes in relation to obesity. The goal is to provide theoretical support for the treatment of other obesity-induced diseases as well as to clarify the mechanisms underlying obesity and its symptoms. 

The body’s ability to maintain homeostasis depends, in large part, on lipid metabolism, and defects in these systems frequently contribute to or worsen the development of obesity. Normally, fats are stored in our bodies as TGs, which lipases hydrolyze to release FAs and glycerol (GI). β-oxidation then gradually breaks down the free fatty acids in mitochondria or peroxisomes. TG hydrolysis is controlled by a mix of substances, enzymes, and hormones. Research indicates that animals on a high-fat diet can create more adrenaline in their visceral adipocytes than mice fed a regular diet [16]. Among these, REEP6 is crucial for β-adrenergic signaling in adipocytes. When REEP6 is inactivated, β-adrenergic signaling is reduced, which reduces energy expenditure and increases obesity. Furthermore, adenylate cyclase 3 (ADCY3) is not expressed in brown adipose tissue (BAT) due to the gene REEP6 KO, which also downregulates REEP6 in adipocytes and decreases plasma membrane-targeted ADCY3 [17]. This enzyme catalyzes the synthesis of ATP to cAMP during the hydrolysis of TGs and also plays a role in mediating the involvement of energy, lipid, and glucose control. Furthermore, studies using cord blood DNA samples and ADCY3-knockout mouse models discovered that increased methylation of genomic DNA sequences can lead to decreased activity of the ADCY3 gene and that decreased expression of the ADCY3 gene at the mRNA and protein levels contributes to obesity [18,19]. Therefore, obesity is made worse by an abnormally low expression of ADCY3 during TG hydrolysis.

G protein-coupled receptors (GPCRs) have been utilized as a major drug target for the treatment of diseases like diabetes mellitus, obesity, AD, and psychiatric disorders because they are crucial links in the adipose hydrolysis process that connect the hormonal stimulation and activation of adenylate cyclase [20]. In a study by Patricio Atanes et al., β-cells in obese individuals’ pancreatic islets were shown to be surrounded by ectopic adipose tissue. They also evaluated the expression of GPCR mRNA. Ectopic adipose tissue enveloping β-cells in obese patients can secrete local adipokines and modify islet GPCR expression and activity through the accumulation of macrophages and the cytokines they secrete [21]. For instance, visceral adipose tissues from obese human donors and subcutaneous fat pads from obese mice both had higher expression of the adherent GPCR family member adhesion G protein-coupled receptor L1 (ADGRL1), and the β-cells in these tissues produced more cAMP. According to this, obese people’s greater insulin secretion is correlated with increased ADGRL1 expression in their pancreatic islets; as a result, reducing ADGRL1 expression may prevent obesity from developing [21,22]. David Ho et al. provided evidence that adenylyl cyclase type 5 (ADCY5) deficiency guards against obesity. They showed that ADCY5 KO mice had significantly lower percentages of body fat and visceral and inguinal fat pads and that their visceral adipocyte size was significantly smaller when they were on a high-fat diet (HFD) [23]. Therefore, the initiation and progression of obesity may be inhibited by blocking ADCY5 activity.

Further research by London, E. et al. revealed that PKA is involved in the regulation of adipose tissue metabolism. PKA also facilitates the binding of hormones, neurotransmitters, and other signaling molecules to the GPCR, which, in turn, controls the levels of cAMP. Through the use of mouse models, it was discovered that diet-induced obesity mice with WAT had decreased cAMP concentrations, PKA and HSL activities, and lipid droplet perilipin phosphorylation. Elevated PKA levels may also regulate lipid metabolism by encouraging lipolysis [24]. The most traditional method of activating the AMPK pathway is PKA signaling, which plays a crucial role in controlling the AMPK pathway. Hanyuan Xu et al. discovered that hordenine could alter lipid metabolism by significantly increasing AMPK phosphorylation, inhibiting adipogenesis (SREBP1, FAS, and ACC), promoting lipolysis (HSL and ATGL), and increasing adipokine expression (FGF21 and ZAG) in the liver and eWAT. Hordenine reduced body weight and fat mass by altering lipid metabolism in mice fed a high-fat diet, while it also increased the energy expenditure of the mice [25]. The body weights of RII KO (PKA RII subunit) mouse pups were lower than those of WT pups following a two-week exposure to an HFD. Furthermore, PKA is expressed differently in important metabolic organs due to the absence of RII subunits, as shown by the increased cAMP-stimulated PKA activity in gonadal adipose tissue but decreased activity in the liver [26]. This implies that targets of PKA signaling may be viable avenues for therapeutic intervention in the context of obesity.

Obesity may also result from abnormal metabolism during fatty acid β-oxidation. A key regulator of β-oxidation, carnitine palmitoyl transferase I (CPTI), in the mitochondria of the liver, heart, and skeletal muscle cells makes it easier for acyl groups to enter the mitochondria for oxidation. The above studies suggested that promoting fatty acid β-oxidation significantly reduces body fat. In animal experiments in rats and mice, when the specific activity of CPTI was effectively increased in skeletal muscle and cardiac tissues, the fat in the epididymis (and viscera) of the experimental subjects was significantly reduced, and the expression of genes related to fatty acid synthesis was suppressed [27]. Further in vivo findings in mice have demonstrated that upregulating CPTI activity stimulates fatty acid oxidation and raises ATP levels, thereby stimulating energy expenditure and reducing body weight in obese mice [28]. Acetyl-coa carboxylase (ACC) is essential for both fatty acid production and fatty acid β-oxidation. FA synthases can employ malonyl-CoA, which is created when ACC catalyzes the carboxylation of acetyl-CoA, to carry out FA biosynthesis. Malonyl coenzyme A is a direct inhibitor of mitochondrial FA absorption and can also decrease CPTI activity [29], which, in turn, inhibits β-oxidation because it is a substrate for FA production. 5 ‘adenosine monophosphate-activated protein kinase (AMPK) controls the metabolism of FAs by blocking FA production and promoting FA β-oxidation via the phosphorylation of decreased ACC activity [30].

### 2.2. T2DM and Abnormal Lipid Metabolism

T2DM is a prevalent metabolic condition characterized by endocrine problems, specifically insulin resistance (IR) [31] and pancreatic β-cell abnormalities [32]. Elevated TG levels, decreased HDL-c levels, delayed catabolism of TG-rich lipoproteins, leading to elevated postprandial TG levels, residual lipoprotein accumulation, and increased LDL production are among the clinical features of abnormal lipid metabolism in T2DM [33,34]. Current studies have shown that obesity is usually a major causative factor of IR [35]. At the same time, IR, during the course of T2DM, will have some negative effects on patients’ lipid metabolism, including dyslipidemia, cholesterol deposition in blood vessels, and cardiovascular diseases like atherosclerosis and coronary artery diseases [33,36,37]. Some studies have found that abnormal cytokines and the recruitment of different types of macrophages (ATMs) are important factors leading to metabolic dysfunction, including T2DM [38,39,40,41]. Additionally, higher plasma levels of both phosphatidylcholines and SMs have been linked to an increased risk of cardiac autonomic dysfunction in recent-onset type 2 diabetes, according to studies [42,43]. Consequently, research on the connection between lipid metabolism and T2DM is very crucial.

Clarifying the connection between aberrant lipid metabolism and IR is crucial for understanding the genesis and progression of T2DM illness since several studies have demonstrated that lipid metabolism abnormalities can further promote T2DM through IR. IR is frequently brought on by abnormalities in lipid metabolism, which impede insulin signaling by accumulating improperly metabolized intracellular lipid mediators and saturated fatty acids, as well as promoting chronic tissue inflammation [44]. Lipid abnormalities and chronic tissue inflammation are closely related. Abnormal lipid metabolism leads to an increase in macrophages (ATMs) in adipose tissue. These ATMs are typically polarized pro-inflammatory M1-like cells [45]. They are recruited by the secretion of different cytokines, including leptin, lipocalin, TNFa, and others [46]. TNFa causes IR through three pathways: paracrine inhibition of IR, paracrine inhibition of IR, and paracrine inhibition of IR. TNFa, IL-1β, leptin, lipocalin, and other cytokines are known to induce IR via three different mechanisms: paracrine inhibition of cells that target insulin, endocrine entry into the internal circulation, and action on the insulin transcriptional machinery to decrease insulin expression [47]. Intracellular specific kinases, receptors, and cytokines that are suitable for the job mediate inflammatory signaling in cells. Pro-inflammatory factors activate their intracellular specific receptors, which raises the expression levels of downstream protein kinases and ultimately triggers the development of inflammation and insulin resistance [35].

Furthermore, through paracrine or endocrine regulatory mechanisms, the miRNAs found in exosomes (Exos) secreted by ATMs can also be targeted to different insulin cell types, with significant effects on cellular insulin action, in vivo insulin sensitivity, and overall glucose homeostasis. According to Jordan et al., obesity increases the production of miR-143 in the liver. By inhibiting its target gene, ORP8, the miRNA reduces the sensitivity of the liver to insulin [48]. Wei Ying et al. discovered that miR-155, which is released by obese mouse ATMs, is absorbed by insulin target cells both in vivo and in vitro. By suppressing the expression of its target gene, PPARg, miR-155 can increase glucose intolerance, systemic insulin resistance, and cellular insulin resistance [49]. Both obese mouse models and human subjects’ livers have been found to express higher levels of miR-802, which inhibits insulin transcription and secretion by driving the Ca^2+^ signaling pathway by encouraging the binding of CREB to the Sox6 promoter [50]. Additionally, miR-802 can reduce insulin sensitivity by blocking the expression of Hnf1b, which also inhibits insulin transcription and secretion [51]. In conclusion, aberrant lipid metabolism can cause IR and an inflammatory response, which can ultimately result in the onset and progression of T2DM.

## 3. CVD and Lipid Metabolism

Globally, there is an exceptionally high death and recurrence rate associated with CVD, a category of illnesses affecting the heart or blood vessels [52,53]. The incidence of abnormal lipid metabolism has been on the rise in recent years due to changes in dietary habits, lifestyle modifications, and improved living standards. Abnormal lipid metabolism is a major risk factor for CVD, particularly atherosclerosis (As) and coronary heart disease (CHD). Anomalous vascular endothelial function and an increased risk of CVD in individuals might result from disorders of lipid metabolism in the blood, which can also impact vasodilatory function and vascular structural remodeling [54]. According to research by Soppert, J. et al., the development of CVD is influenced by the buildup of LDL-C, HDL-C, lipoproteins, TG, FAs, and derivatives [55]. Among them, reducing plasma apoB, which comprises lipoproteins, has been thought to be crucial for both treating and preventing CVD since it reduces the incidence and progression of the disease [56]. Furthermore, research has demonstrated that during obesity, hormones produced from adipose tissue, such as resistin and leptin, are harmful to cardiovascular health [6,7,8]. Many CVDs, such as coronary heart disease, heart failure, hypertension, stroke, atrial fibrillation, and sudden cardiac death, have been linked to abnormalities in lipid metabolism. These abnormalities can also either directly or indirectly raise the morbidity and mortality rates associated with cardiovascular diseases [52,53,54,55,56,57].

### 3.1. Arachidonic Acid Metabolic Pathway, Abnormal Metabolism, and CVD

According to a recent study, TGs and polyunsaturated fatty acids (PUFAs) are important in the prevention and treatment of CVD. Lowering TG levels has been shown to lessen the risk of CVD [58]. Arachidonic acid (AA) is a long-chain polyunsaturated omega-6 fatty acid that is very prevalent in the human body. A plethora of research conducted in the past few years has indicated that AA is crucial for cardiovascular health. Since CVD affects the inner lining of the arteries, it is a chronic inflammatory disease. It is primarily brought on by abnormalities in lipid metabolism and the buildup of macrophages that contain cholesterol in the arterial walls. These events trigger an adverse immune response in the body, which typically manifests as plaques in medium- and large-sized arteries [59], which causes the arteries to become stiffer and less elastic [60]. Several metabolites in the AA metabolic pathway have been demonstrated in several studies to influence cardiovascular disorders like As. Certain AA metabolites generated by cytochrome P450 (CYP), arachidonic acid lipoxygenases (ALOXs), and prostaglandin-endoperoxide synthase (PTGS) are significant contributors to the development and incidence of CVD in the AA metabolic pathway [61]. For instance, phospholipase A2 liberates phospholipids from cell membranes as AA, which is then processed by three separate enzymes, PTGS, ALOX, and CYP, and enters three distinct metabolic pathways. Because it influences the key pathophysiological elements of As and ischemic heart disease, such as platelet aggregation, arterial wall tone, and inflammatory processes in motile As lesions, the PTGS pathway is one of the most important therapeutic targets in both conditions. Prostaglandins (PGs), prostacyclin (PGI2), thromboxane A2 (TXA2), hydroxye icosatetraenoic acids (HETEs), leukotrienes (LTs), lipoxins (LXs), and epoxyeicosatrienoic acids (EETs) are among the products that are produced by AA’s three metabolic pathways [62]. These products all modulate vascular tone by acting on various receptors, and each is crucial in cardiovascular complications [63]. For example, the metabolites PGD2 [64,65,66], PGF2 [67], and PGI2 [68] in the PTGS pathway inhibit thrombosis by lowering blood pressure and vascular tone to cause vasodilatation, whereas the products PGE2 [69,70,71,72,73,74] and TXA2 [75,76,77] have the opposite effect. In the ALOX pathway, the metabolites LTs play a role in promoting vasoconstriction and thrombosis [78,79], while another product, LXs, has an inhibitory effect on thrombosis, in contrast [80,81,82]. The HETEs in the third CYP pathway, which play their respective roles in the vasculature according to their different isomers, are noteworthy [83,84,85,86] (Table 1). Therefore, from the perspective of regulating lipid metabolism, it is important to explore new therapeutic targets and screen new drugs for the treatment of cardiovascular diseases. 

### 3.2. Abnormal Lipoprotein Metabolism and CVD

A significant cause of CVD during lipid metabolism is generally thought to be the buildup of low-density lipoprotein cholesterol (LDL-C) as a result of metabolic disorders [88,89,90]. Several investigations have demonstrated that As can be successfully prevented from occurring and developing by directly inhibiting LDL transport. Antibodies derived from sulfated glycosaminoglycans in proteoglycans were discovered by Soto et al. to impede the retention of LDL, which might lessen LDL modification and prevent the growth of As [91,92]. In a study conducted by Sessa et al., mice deficient in activin receptor-like kinase 1 (ALK1) in the arterial endothelium (Alk1^iΔaEC^) were crossed with mice devoid of the LDL receptor (Ldlr^−/−^). Compared with the control group, the crossbred mice showed significantly less As plaque formation, ApoB, and macrophage infiltration [93], confirming the notion that obstructing LDL transport inhibits As development.

**Figure 1:**
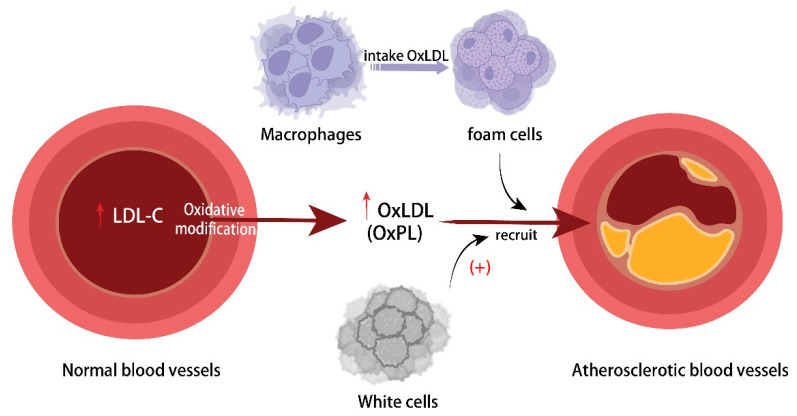
Mechanism of atherosclerosis caused by abnormal lipid metabolism.

LDL may be modified in a number of ways, the most common being oxidation, which can result in As [4]. Other alterations include glycation and oxidative modification. Oxidized low-density lipoprotein (Ox-LDL) resulting from oxidative modifications, such as oxidized phospholipids (OxPLs), can be ingested by macrophages, which subsequently transform into foam cells [94,95]. The build-up of foam cells increases the risk of lipid streaks and even lipid plaques, which ultimately cause CVD to occur and progress. Furthermore, the build-up of cholesterol within macrophages stimulates inflammatory reactions, which, in turn, triggers the activation of the NLRP3 inflammasome through NF-κb-mediated activation, the production of pro-inflammatory cytokines, and the aggravation of the chronic inflammatory state of atherosclerosis [96]. This also damages the vascular endothelium and increases the expression of adhesion molecules [97], which, in turn, triggers more macrophage aggregation and matrix metalloproteinase (MMP) expression, diminishing the stability of As plaques and aggravating the spread and development of As plaques [98,99]. Shi et al. found that BAT-derived neuromodulin-4 (neuregulin-4 (Nrg4)) inhibited Ox-LDL-induced inflammatory responses (TNF-α, IL-1β, and IL-6) and the migration of RAW264.7 cells. In vivo, Nrg4 was shown to reduce endothelial damage and ameliorate the onset and progression of As in mice [100]. In experimental and in vitro studies in HFD-fed mice, Gang Luo and colleagues found that inhibiting p38 MAPK phosphorylation and p16 expression in the p38 MAPK/p16 pathway dramatically decreased Ox-LDL-induced macrophage senescence and reduced the development of As [101]. Furthermore, glycated low-density lipoprotein (G-LDL), which is produced when LDL glycation is modified, stimulates macrophage foam cell formation, increases oxidative stress and inflammation in human endothelial cells, and binds to scavenger receptor A (SR-A) to upregulate SR-A gene expression and extend its half-life, which stabilizes the transfer of more G-LDL and exacerbates the As outbreak [102]. As a result, anomalies in lipid metabolism cause LDL to aggregate underneath the endothelium in the artery vasculature, which aids in the onset and advancement of As (Figure 1).

As the enzyme converts citric acid to acetyl coenzyme A, it offers precursor material for the subsequent production of fatty acids and cholesterol. This makes the study-confirmed ATP citrate lyase (ACLY) an attractive target for LDL-C reduction and cardiovascular protection. In fact, ACLY uses distinct pathways to target macrophages and hepatocytes in order to achieve its dual regulatory actions [103,104]. In an animal model of As, Pinkosky et al. discovered that using benzoic acid to suppress ACLY expression in hepatocytes was successful in lowering circulating LDL-C levels [105]. After inhibiting ACLY activity with the inhibitor 326E, Zhifu Xie et al. upregulated LDLr expression and discovered that apolipoprotein E (ApoE)-deficient mice had significantly fewer As plaques, both in terms of number and size, as well as the expression of inflammation-associated genes Cd68, F4/80, and Il1b, as well as ATP-binding cassette subfamily G member 5 and 8 (ABCG5 and ABCG8) genes, which are linked to hepatic cholesterol efflux [106]. This suggests that the anti-As blocked ACLY functions through hepatic cholesterol efflux. Meanwhile, lipopolysaccharide (LPS) and IL-4-induced macrophage activation are modulated by ACLY-dependent acetyl coenzyme A doping of histones, which mostly exacerbates As by boosting inflammatory responses. In vitro studies showed that blocking ACLY decreased oxPAPC, a stimulus linked to in vivo atherosclerosis, as well as LPS-induced IL-1β synthesis and hypoxia-inducible factor (HIF)-1α signaling, which, in turn, decreased macrophage inflammatory responses [107]. Pathological analysis of the As plaques in AclyM-KO mice revealed increased atherosclerosis of the plaques’ thick fibrous cap and significantly smaller areas of plaque necrosis. Additionally, the Acly^M-KO^ mice’s macrophages had elevated levels of transforming growth factor β (TGF-β), indicating that ACLY exerts its effects on As through the expression of TGF-β by macrophages [108]. As a result, As can be successfully prevented from occurring and from developing when ACLY activity in lipid metabolic pathways is inhibited. This makes ACLY an important target for possible As therapy.

Apart from the issues with low-density lipoprotein (LDL), new research has demonstrated that high levels of triglyceride-rich lipoprotein residues (TRLs) derived from the gut and liver are linked to cardiovascular disorders such As [109,110]. Newly secreted TRLs in chylomicron (CM) undergo intravascular lipolysis to produce chylomicron remnants, which, in turn, yield a variety of highly modified particles in very low-density lipoproteins (VLDLs), including remnants in the VLDL density range, intermediate-density lipoproteins (IDLs), and LDLs. These smaller, highly modified TRL remnant particles are becoming more widely acknowledged as crucial As therapeutic targets [109]. Moreover, Bäck et al. discovered that FAs can control intimal inflammation in a variety of ways [59]. Omega-3 polyunsaturated fatty acids (w3 PUFAs) interact with free fatty acid receptor 4 (FFAR4) to attenuate vascular inflammation, arterial thrombosis, and intimal hyperplasia, thereby acting as a vascular repair agent. For instance, omega-3 fatty acids themselves can directly activate specific free fatty acid receptors (FFARs) [111,112]. However, fatty acid-derived peroxidation products, like lipoaldehyde 4-hydroxy-nonenal (HNE), can cause plaque instability and rupture by forming HNE-apoB adducts. These adducts can divert LDL metabolism from pathways that allow macrophages to scavenge receptors, which can result in the formation of foam cells and may also locally cause apoptosis in smooth muscle cells and macrophages. Ultimately, this can cause plaque instability and rupture and raise the risk of atherosclerotic thrombotic events [113,114]. In conclusion, As is mostly regulated by lipid metabolism, and more research into this area should lead to improved As symptoms through lipid metabolism interventions.

Sphingolipids are a highly diverse and complex structure of lipid families, widely distributed in cell membranes or plasma lipoproteins, including ceramide (Cer), sphingosine (So), sphingomyelin (SM), sphingosine-1-phosphate (S1P), and glucosylceramide. With the advancement of plasma lipidomics, more and more researchers have recognized the role of sphingomyelins in the onset and progression of cardiovascular disease. The risk of CVD was associated with circulating ceramides and sphingomyelin, which changed depending on the length of connected acylated saturated fatty acids [115,116]. Heart failure was associated with greater plasma levels of Cer-16 and SM-16, but not with higher levels of Cer-22 or SM, a very-long-chain saturated fatty acid [115]. A study of older persons in the Cardiovascular Health Study (CHS) indicated that higher levels of plasma Cer-16 or SM-16 increased the risk of death, while lower levels of longer fatty acid species decreased the risk of mortality [116]. Higher levels of plasma Cer, particularly Cer-16, have been linked to an increased risk of diabetes, as well as an increased risk of cardiovascular disease after diabetes development [117]. The plasma concentrations of Cer-16 and SM-16 were linked to an increased risk of sudden cardiac death (SCD) [118]. Currently, the beneficial processes of a greater length of acylated saturated fatty acid coupled to Cer and SM are unknown. Therefore, future research is needed to investigate the beneficial processes of longer acylated saturated fatty acid chains coupled to Cer and SM.

## 4. Abnormalities of Lipid Metabolism in Neurodegenerative Diseases

Major constituents of neuronal cells in the brain, lipids perform both structural and physiological roles in processes such as neural communication, neurogenesis, synaptic transmission, signal transduction, membrane region compartmentalization, and gene expression control. Neurodegenerative illnesses including Alzheimer’s and Parkinson’s have been shown to exhibit dysregulation of certain lipid classes and lipid homeostasis. Deficits in emotion control, stress management, and learning and memory are caused by experimental suppression of neural stem cell (NSC) activity in the brain [119]. Lipids’ function in controlling NSC behavior has drawn more attention in the last several years. Lipid metabolism genes represent a prominent category of transcriptional variations between resting and active NSCs in the adult subventricular zone (SVZ) ecotope [120]. Moreover, fatty acid oxidation is necessary for neural precursors to proliferate in the SVZ and hippocampal dentate gyrus (DG) ecotopes [121], whereas NSCs are found in the SVZ and are especially sensitive to ambient lipid signals. It has been discovered that monotherapy with omega-3 unsaturated fatty acids (N-3 PUFAs) slows down indications of neurodegenerative illnesses, such as decreased practice and spoken language skills [122].

Alzheimer’s disease (AD) is a chronic, progressive neurological illness that has garnered a lot of attention in recent years [123]. Patients with AD may have lipid metabolic abnormalities, including TG, TC, LDL-C, apolipoprotein B (ApoB), apolipoprotein E (ApoE), etc. Thus, the onset of AD is intimately linked to brain lipid metabolite abnormalities [124]. Arachidonic acid (AA), acrylic acid (ALA), eicosapentaenoic acid (EPA), and docosahexaenoic acid (DHA) are examples of polyunsaturated fatty acids (PUFAs) that have been shown to have anti-inflammatory or pro-inflammatory effects as well as some neuronal protective properties [125]. For example, DHA and EPA have been shown to decrease the secretion of pro-inflammatory cytokines by increasing the production of growth factors like neurotrophic factors, which, in turn, helps AD [126].

Endocannabinoids are lipophilic compounds containing long-chain polyunsaturated fatty acids that can bind to and activate cannabinoid receptors such as CB1 and CB2 [127]. Cannabinoid (CB) receptors are G protein-coupled receptors found throughout the body, including the central and peripheral neurological systems [128]. Under pathological settings, CB1 receptors are more common and more upregulated in microglia than CB2 receptors, and binding to endocannabinoids or phytocannabinoids can trigger microglia to change into the less damaging M2 phenotype and protect neurons [127,129]. A recent study demonstrated that endocannabinoids N-arachidonoyl ethanol amide and noladin directly interact to hinder Aβ42 self-assembly [130]. AppNL-G-F mice with APP knock-in showed enhanced expression of CB2 receptors at 6 and 12 months of age, as well as GPR55 mRNA levels and immunoreactivity [131].

Parkinson’s disease (PD), Parkinson’s disease dementia (PDD), and other neurological diseases are included in the category of dementia with Lewy bodies (DLB) disorders [132]. The risk of developing Parkinson’s disease is influenced by dietary fat consumption. A high intake of saturated fats, such as cholesterol and arachidonic acid, may raise this risk, while a reduced intake of saturated fats may be advantageous for the prevention of Parkinson’s disease [133,134]. Maintaining the homeostasis of organismal lipid metabolism is also crucial, as evidenced by the discovery that neuropeptidergic neurons exhibit excessive endoplasmic reticulum–mitochondrial contact sites during disrupted early sleep patterns in Parkinson’s patients [135]. These excessive contact sites cause aberrant lipid transport, depletion of phosphatidylserine in the ER, and disruption of neuropeptide-containing vesicle production. Functional studies have revealed that the wild-type PTEN-induced putative kinase 1 (PINK1) protein may protect nerve cells from stress-induced mortality [136]. A PINK1 gene mutation may cause Parkinson’s disease. CB1 receptors in homozygous PINK1^−/−^ mice drastically impair corticostriatal glutamatergic synaptic transmission ability [137]. Compared with the control group, patients with Parkinson’s disease had significantly higher mRNA levels of CB1 receptors [138].

## 5. Abnormalities in Lipid Metabolism in Cancer

One of the main indicators of the onset of cancer is abnormal lipid metabolism in cells. Lipid metabolism disorders frequently affect a range of signaling pathways involved in cancer genesis, invasion, and metastasis [2,3,4,5,6,7,8]. Understanding the mechanism of aberrant lipid metabolism in cancer cells in order to target and control the expression of key genes in the lipid metabolic pathway is anticipated to be a new approach to cancer treatment, aided by extensive domestic and international research. The development of cancer is significantly influenced by lipid metabolism [139], and problems with lipid metabolism frequently result in the aberrant expression of several genes and proteins as well as the dysregulation of cytokines and signaling cascades. 

### 5.1. Abnormalities in Lipid Metabolism in Tumor Cells

Rapid cancer cell proliferation is a key step in the process of cancer growth, and the FA continuous composite membrane and signal molecules are required to support cancer cell proliferation [140]. Consequently, it is important to investigate the impact of lipid metabolism on cancer formation by examining how FA production and fatty acid oxidation (FAO) affect cancer cell metabolism. Simultaneously, elevated levels of β-oxidation and FA de novo synthetic production have been demonstrated in cancer cells. And these noticeably raised properties have been confirmed in several cancer types [141,142].

During the synthesis of endogenous lipids in cancer cells, FAs are synthesized from a cytoplasmic acetyl coenzyme produced from glucose, glutamine, or acetate, and subsequently undergo a condensation step catalyzed by acetyl coenzyme a carboxylase (ACC1/2, also known as ACACA/B), malonyl coenzyme a, and fatty acid synthase (FASN) to form 16-carbon saturated FA palmitate [143], and finally, palmitate forms non-essential fatty acids including 18-carbon monounsaturated fatty acids (C18:1) by the action of fatty acid elongase of very-long-chain fatty acids 1–7 (ELOVL1-7), stearoyl coenzyme a desaturase, or fatty acid desaturase 1–3 (FADS1-3), with the key enzymes involved in this process all playing an important role in the development of cancer [144]. Sphingolipids (glucose ceramides) and glycerophospholipids (cardiolipins) are synthesized in response to mTORC2 stimulation during hepatocellular carcinogenesis. This process further amplified mTORC2 activity and caused hepatocellular carcinoma (HCC) in a rat model [145]. Similar to this, in ovarian cancer cells, fatty acid synthesis and the phosphorylation of the NF-κB signaling pathway are inhibited by blocking FASN and carnitine palmitoyl transferase 1A (CPT1A) activity through miR-33b’s targeting of growth factor β-activated kinase 1 (TAK1), which, in turn, prevents the peritoneal metastasis of ovarian cancer [146]. In prostate cancer cells, xenografts, and clinical tumors, the direct activation of androgen receptor (AR)-mediated fatty acid elongase of very-long-chain fatty acid 5 (ELOVL5) has been shown [147]. Moreover, it was discovered that the MAPK pathway’s MEK5-ERK5 is necessary for the survival and growth of SCLC cell lines both in vivo and in vitro and that its loss compromises lipid metabolism pathways like the mevalonate pathway, which regulates the synthesis of cholesterol [148]. 

In addition, sphingolipid metabolism abnormalities are commonly detected in several kinds of cancer [149,150,151]. Ceramide kinase (CERK) is a sphingolipid metabolic enzyme that can phosphorylate intracellular ceramide and, hence, promote cancer cell growth [152]. Upregulating CERK expression levels may prevent tamoxifen-induced Cer accumulation, which is related to a poor prognosis in estrogen receptor-positive breast cancer patients receiving endocrine therapy [153]. As the S1P/Cer ratio grows, tumor cell survival and/or proliferation increase, boosting colorectal cancer progression while decreasing chemotherapy sensitivity [154]. Exosomes, a potential non-invasive cancer biomarker, are gaining popularity among researchers. A study discovered that nine lipid species in urine exosomes were significantly different between a prostate cancer patient group and a healthy group [151]. According to the studies mentioned above, several classes of sphingolipids may be raised in certain human malignancies, and sphingolipid metabolic abnormalities may contribute to the occurrence and development of cancer.

During lipid metabolism, monoacylglycerol lipase (MAGL) transforms into free fatty acids and glycerol. In tumor cell lines, MAGL gene expression and protein levels rise with tumor cell malignancy [155]. MAGL exhibits high levels of expression in invasive human carcinomas and primary tumors. Through the upregulation of pro-tumor-signaling lipids in tumor cell lines [156], as well as the increased expression of its protein and gene [155,156,157,158], MAGL facilitates tumor invasion and metastasis. After that, fatty acids go into the β-oxidation phase. In cancer biology, β-oxidation has drawn a lot of interest as one of the key mechanisms to stop tumorigenesis and development. Gloria Pascual and colleagues showed that the fatty acid receptor CD36, by increased lipid absorption and activating β-oxidation, increases the development of human oral carcinomas by increasing oral carcinogenesis in a mouse model of oral squamous cell carcinomas (OSCCs) [159]. Hepatocellular carcinoma (HCC) cells have been found to significantly under-express medium-chain acyl-coenzyme a dehydrogenase (ACADM), an enzyme that catalyzes the first step of mitochondrial fatty acid oxidation. When caveolin-1 (CAV1) expression is increased, this leads to the nuclear accumulation of SREBP1, which inhibits ACADM activity and, as a result, fatty acid oxidation, and increases the invasiveness of HCC cells [160]. In MNA neuroblastoma, the fatty acid metabolism process is promoted, and lipid metabolism becomes unbalanced due to the increased expression of ACADM and the inhibition of aurora kinase A (AURKA) and aurora kinase B (AURKB) [161]. Furthermore, in clear-cell renal-cell carcinoma (ccRCC), there is an increased expression of stearoyl coenzyme a desaturase 1 (SCD1), fatty acid synthase (FASN), and acetyl coenzyme a carboxylase (ACC). This results in the formation of abnormal pathways that produce high levels of acetyl coenzyme a and fatty acids [162]. In conclusion, aberrant lipid metabolism found in cancer cells is closely linked to the development of cancer. 

### 5.2. Abnormalities in Lipid Metabolism in the Tumor Microenvironment

Cancer cells regulate their own metabolism, including lipid metabolism, in order to adapt to the changes in the tumor microenvironment (TME) that are typically associated with the occurrence and progression of malignant tumors [163]. These changes are characterized by aberrant changes in metabolic signals, lipid transport proteins, metabolic substrates, metabolic enzymes, and metabolites in lipid metabolism [140], which are primarily manifested as abnormal accumulation of lipids in tumor cells [164]. Abnormal lipid accumulation in the tumor microenvironment can influence the phenotype and functionality of immune cells that infiltrate tumors, which helps create an immunosuppressive tumor microenvironment and allows tumor cells to evade the immune system. Stearoyl-CoA desaturase 1 (SCD1), a rate-limiting enzyme, is involved in the conversion of saturated fatty acids into monounsaturated fatty acids (MUFAs), and it is a key indicator of the tumor microenvironment. Because SCD1 is expressed at high levels in a number of cancer types and regulates fatty acid metabolism, it has been proposed as a possible target for cancer therapy [165]. It has been shown that these SCD1-related signals activate the β-catenin pathway, which has been linked to inflammatory tumors that are not T-cell-related in a range of human malignancies, such as melanoma, colorectal, and hepatocellular cancers [166]. Prostate cancer and other tumor types have also been shown to have upregulated SCD1 expression or activity, which may aid in the development of cancer by stimulating the AKT signaling pathway or by blocking AMPK and GSK3, which, in turn, promotes downstream β-catenin activity and triggers associated tumor growth signals [167]. The research conducted by FRITZ et al. provided additional confirmation that pharmacological inhibition of SCD1 activity reduces lipid synthesis, inhibits the growth of androgen-sensitive and androgen-resistant prostate cancer cells, stops the growth of prostate tumor xenografts in nude mice, and improves survival in nude mice [168]. Furthermore, CYH33, an inhibitor of the phosphatidylinositol 3-kinase (PI3K) pathway, stimulates FA metabolism, raises free FA (FFA) levels, and activates CD^8+^ T cells in the TME. These actions prevent tumor development and improve host immunity [169]. Lipid lung mesenchymal cells (MCs) use exosome-like vesicles to transfer their lipids to tumor cells and natural killer (NK) cells. MCs specifically alter mouse adipose triglyceride lipids (ATGLs), which increases tumor cell proliferation, impairs NK cell function, and increases lung metastasis from breast cancer [170]. Therefore, control of lipid metabolism in the tumor microenvironment frequently significantly contributes to the initiation and spread of cancer. 

## 6. Lipid Metabolism Abnormalities in Other Diseases

The capacity to control lipid metabolism is essential for sustaining health since it is a complicated physiological process that is intimately tied to dietary management, hormone regulation, and homeostasis. However, severe lipid metabolism abnormalities are a result of long-term dietary excess and bad lifestyles in modern culture. Chronic illnesses including polycystic ovaries, osteoarthritis, chronic kidney disease (CKD), nonalcoholic fatty liver disease (NAFLD), and others frequently coexist with lipid metabolism abnormalities. 

Hepatic steatosis results from increased hepatic fat buildup combined with impaired hepatic lipid clearance, and non-alcoholic fatty liver disease (NAFLD) is intimately linked to changes in lipid metabolism [171]. The lipid metabolism transcription factor FOXO1 has a dual regulatory function in nonalcoholic fatty liver disease (NAFLD); overexpression of this factor in the liver can result in increased TG production, decreased hepatic lipid oxidation, and worsened lipoatrophy. The majority of the current research on NAFLD prevention and therapy has focused on disrupting the disease’s lipid metabolism signaling system. For example, Yue Li et al. [1] negatively regulated the transcription factor SREBP1, which causes fat deposition and ameliorates NAFLD symptoms. According to Chen et al. [171], hypericin (HP) directly binds to PKACα, triggering the PKA/AMPK signaling cascade to produce its regulatory effects on non-alcoholic fatty liver disease. Furthermore, studies have discovered that a variety of food extracts can target hepatic fatty acid degeneration by triggering the corresponding lipid metabolism signaling pathways. For example, the extract cochineal orange Bixin functions as a Nrf2 activator, which targets oxidative inflammation and hepatic steatosis [172]; meanwhile, it can activate PPARα, which, in turn, triggers hepatic fatty acid oxidation, improving lipid and carbohydrate metabolism in obese mice [173].

In the kidneys, glomeruli function as selective filters, eliminating harmful substances from the blood. Chronic kidney illness is frequently the result of glomerulus lesions, and podocytes functionally specialized, terminally differentiated cells of the glomerular filtration barrier are highly susceptible to lipotoxicity [174]. Diabetic kidney disease (DKD) is mostly determined by lipid buildup in podocytes; hence, it is now crucial, from a clinical standpoint, to identify possible treatment targets by modulating podocyte lipid metabolism [175]. Through the SIRT1-mediated SREBP1 signaling pathway, JAML causes enhanced lipid accumulation in podocytes when junctional adhesion molecules (JAMs) are overexpressed in the cells. According to Ming Wu et al. [176], treatment of inflammatory vesicles enriched with nucleotide-containing leukocyte polypeptide 3 (NLRP3) siRNA significantly inhibited the production of mitochondrial ROS in podocytes, cytoskeletal changes, lipid accumulation, and high-glucose-induced apoptosis. It has been demonstrated that lipotoxicity-induced podocyte damage results from the dysregulation of lipid metabolism, which raises intracellular free fatty acid levels or accumulates cholesterol and is linked to mitochondrial dysfunction [177].

The most frequent joint condition that affects a large percentage of the senior population is osteoarthritis (OA) [178]. Proteomic analyses have revealed a significant relationship between OA and lipid metabolism, with research showing that adipokines are important regulators in the pathophysiology of OA [179] and that disorders of lipid metabolism and low-grade inflammation have a greater impact on joint tissues [180]. Meanwhile, n-3 PUFAs increase osteoblast production by downregulating PPARγ and boosting osteoblast activity, n-6 PUFAs limit osteoblast differentiation by raising the expression of peroxisome proliferator-activated receptor gamma (PPARγ) and encouraging adipose formation [181,182]. Through the inhibition of mTORC1 and the promotion of chondrocyte autophagy and cell survival, greater synthesis of n-3 PUFAs from endogenous n-6 PUFAs may postpone the beginning of OA, according to the results of mice research [183]. Prostaglandin E2 (PGE2), a metabolite of arachidonic acid (AA), and its own nuclear factor kappa-B ligand (RANKL) pathway receptor activators are important regulators of osteoblast development [184]. It was shown that modest doses of omega-3 polyunsaturated fatty acid supplementation were adequate to mitigate the effects of obesity on osteoarthritis (OA) and aided in expediting OA healing in mouse tests including dietary fatty acid supplementation. Meanwhile, increased bone mineral production, heterotopic ossification, synovitis, and enhanced macrophage infiltration in synovial tissue were all consequences of osteoarthritis following joint damage caused by SFA and ω-6 PUFA supplementation, respectively [185].

## 7. Summary

**Figure 2:**
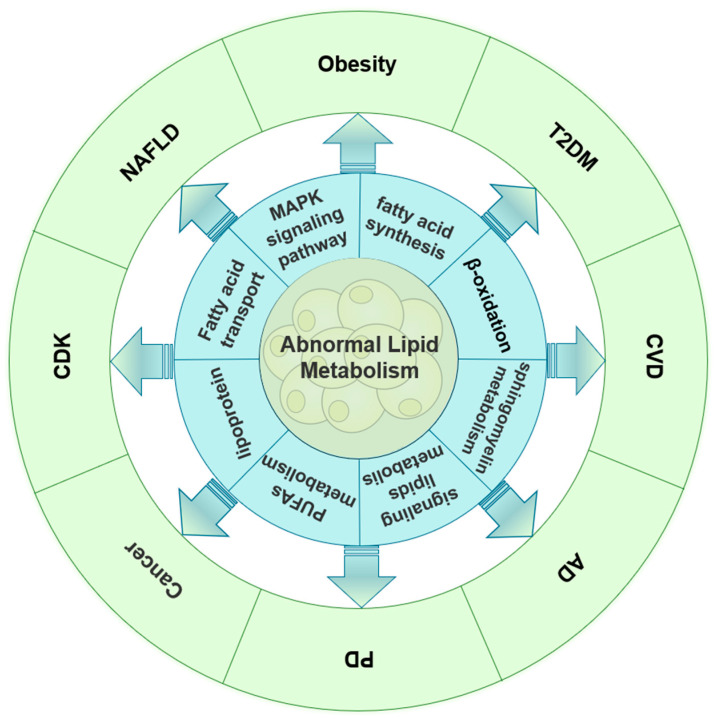
Lipid metabolism disorders and various diseases.

As the study of lipid metabolism continues, anomalies in this process are becoming more and more linked to the onset and progression of many illnesses. In order to comprehend the role of lipid metabolism in the pathogenesis of various diseases and ultimately achieve the goal of the effective prevention and treatment of related diseases, researchers are currently concentrating on investigating the association mechanism between lipid metabolism and various related diseases. The dynamic equilibrium of lipid metabolism in the body is achieved by the combined activity of several genes and metabolic enzymes. Lipid metabolism is a complicated metabolic process that takes place in several organ groups and at different levels. It is anticipated that research on important lipid metabolism genes or enzymes as therapeutic targets may yield new insights into the etiology and management of associated disorders. The application of lipidomics in the exploration of various disease markers is still in its infancy. There are also few studies on the role of lipid metabolism-related genes in the disease, the specific biological mechanisms, and the related key signaling pathways. This review focused on the role that aberrant lipid metabolism plays in the development of several illnesses, including cancer, neurological disorders, endocrine system diseases, and cardiovascular system diseases (Figure 2). Thus, the relationship between the abnormal expression of more key lipids, lipid molecules, key lipid metabolism genes, and key lipid metabolism enzymes and the occurrence and development of various diseases has been further clarified. It also provides a new perspective for the efficient prevention, diagnosis, treatment, and mechanism research of diseases related to abnormal lipid metabolism.

## Figures and Tables

**Table 1:** Metabolic pathways of arachidonic acids in the action of three pathways.

Pathways	Metabolites		Receptors	Action on Blood Vessels	Vascular State	Reference
Prostaglandin-endoperoxide synthase (PTGS)	Prostaglandins (PGs)	PGD2	DP1; DP2	Vascular tension (−); blood pressure (−)	Vasodilation; thrombus (−)	[64,65,66]
		PGE2	EP1; EP3	Vascular tension (+); blood pressure (+)	Vasoconstriction; thrombus (+)	[69,70,71]
			EP2; EP4	Vascular tension (−); blood pressure (−)	Vasodilation; thrombus (−)	[72,73,74]
		PGF2α	FP	Vascular tension (+); blood pressure (+)	Vasoconstriction; thrombus (+)	[67]
	Prostacyclin (PGI2)		IP	Vascular tension (−); blood pressure (−)	Vasodilation; thrombus (−)	[68]
	Thromboxane A2(TXA2)		TP	Vascular tension (+); blood pressure (+)	Vasoconstriction; thrombus (+)	[75,76,77]
Arachidonic acid lipoxygenases (ALOXs)	Leukotrienes (LTs)		-	Vascular tension (+); blood pressure (+)	Vasoconstriction; thrombus (+)	[78,79]
	Lipoxins (LXs)		-	Vascular tension (−); blood pressure (−)	Vasodilation; thrombus (−)	[80,81,82]
Cytochrome p450 (CYP) enzymes	Hydroxye icosatetraenoic acids (HETEs)		-	Different HETEs have different effects on blood vessels		[83,84,85,86,87]
	Epoxyeicosatrienoic acids (EETs)		-	Vascular tension (−); blood pressure (−)	Vasodilation; thrombus (−)	[63]
